# Quantitative and simplified [18F] fluoroestradiol positron emission tomography (PET) measures of brain estrogen receptor expression

**DOI:** 10.1007/s00259-025-07470-1

**Published:** 2025-08-07

**Authors:** Matilde Nerattini, Valentina Berti, Dawn C. Matthews, Schantel Williams, Caroline Andy, Francesca Fauci, Camila Boneu, Trisha Ajila, Silky Pahlajani, Michael Battista, Randolph Andrews, Alberto Pupi, Joseph R. Osborne, Matthew Fink, Roberta Diaz Brinton, Jonathan P. Dyke, Lisa Mosconi

**Affiliations:** 1 Department of Neurology, Weill Cornell Medicine, New York, NY USA; 2 Department of Biomedical Experimental and Clinical Sciences “Mario Serio”, University of Florence, Viale Morgagni, 50, Florence, 50134 FI Italy; 3 ADM Diagnostics, Northbrook, IL USA; 4 Department of Population Health Sciences, Weill Cornell Medicine, New York, NY USA; 5 Department of Radiology, Weill Cornell Medicine, New York, NY USA; 6 Department of Pharmacology and Neurology, University of Arizona, Tucson, AZ USA; 7 Weill Cornell Neurology, 402 East 70th Street, New York, NY 10021 USA

**Keywords:** Fluoroestradiol (FES), Positron Emission Tomography (PET), Logan plot, Brain, Estrogen receptors, Women

## Abstract

**Purpose:**

Positron emission tomography (PET) with 16α-[^18^F]fluoro-17β-estradiol (^18^F-FES) allows for the in vivo assessment of brain estrogen receptor (ER) expression. This study examines brain ^18^F-FES uptake to define an optimal acquisition time for static late images suitable for clinical application.

**Methods:**

Fifty-five healthy, 40–65-year-old women at different endocrine aging stages (*n* = 18 premenopause, *n* = 18 perimenopause, and *n* = 19 postmenopause) underwent dynamic 90-minute ^18^F-FES PET imaging. We obtained regional brain distribution volume ratios (DVR) based on Logan graphical analysis and standardized uptake value ratios (SUVR) at five 20 min increments (30–50, 40–60, 50–70, 60–80 and 70–90 min post-injection), using the cerebellar gray matter as the reference. We used reliability analysis and automated variable selection procedures to identify the most consistent SUVR time windows relative to DVR. In sensitivity analyses, we tested for group differences and associations with cognitive performance in these SUVR time frames. Analysis focused on the pituitary gland, which has demonstrated specific binding. Exploratory ER-rich regions of interest (ROI) included hypothalamus, hippocampus, amygdala, caudate, frontal and cingulate cortex.

**Results:**

SUVR measurements exhibited stronger associations with DVR at earlier compared to later time frames. Specifically, the optimal SUVR time frames in pituitary, and in most exploratory ROIs, were predominantly within the 30–50 and 40–60 min intervals. Both intervals were effective at differentiating postmenopausal versus premenopausal groups, and the 30–50 min window showed more significant associations with cognitive scores.

**Conclusions:**

Examination of quantitative and simplified methods for analysis of brain ^18^F-FES PET uptake identified the 30–60 min SUVR window as performing optimally relative to DVR measures. This provides a practical method for quantifying relative pituitary tracer retention in clinical populations.

## Introduction

17β-estradiol (E2), the most potent estrogen hormone in the circulation, is a gonadal sex steroid hormone that exerts wide-ranging effects on neurological and cognitive functions [1–3], influencing both neurodevelopmental and neurodegenerative processes [2, 4]. E2 acts through estrogen receptors (ERs) ubiquitously distributed in brain [1, 2] where they orchestrate neuroprotective signaling pathways [5] and influence blood flow, energy metabolism, inflammation, and oxidative processes [6, 7].

All women experience a reduction in circulating E2 as they undergo menopause – a midlife neuroendocrine transition state which culminates with reproductive senescence [8]. Menopause-related E2 declines and changes in ER regulation [5] are associated with neurological symptoms including vasomotor symptoms, low mood, disturbed sleep and cognitive changes [4, 9], as well as an increased risk of brain injuries, mood disorders, parkinsonism, and Alzheimer’s disease (AD) [10].

Currently, positron emission tomography (PET) with selective ER ligands is the only technique that allows for the in vivo assessment of ER expression. The ligand 16α-^18^F-fluoro-17β-estradiol (^18^F-FES) is the most utilized ER ligand in oncology, exhibiting selective binding affinity for ERs [11], particularly ER alpha expression [12], as confirmed through immunohistology and biochemical receptor assays [11, 13, 14].

Preclinical work indicated the feasibility of applying ^18^F-FES to brain studies. In female rodents, specific ^18^F-FES binding was reported in ER-rich brain regions such as pituitary and hypothalamus [15–17]. Lower yet measurable signal was observed in preoptic area, hippocampus, striatum, amygdala and cortex [15–18]. Specific pituitary binding was also reported in the rhesus macaque brain [19]. ^18^F-FES uptake in pituitary [16, 17] and hypothalamus [16] was increased in ovariectomized rats, possibly reflecting ER upregulation in response to estrogen deprivation.

To date, three brain ^18^F-FES PET studies have been conducted in women. One whole-body PET study examined pituitary uptake in a cohort of breast cancer patients, showing trends towards higher standardized uptake value ratios (SUVR) in postmenopausal compared to premenopausal patients [17]. Another study – a kinetic analysis of seven healthy postmenopausal women - demonstrated the feasibility of ^18^F-FES PET imaging for assessing pituitary ER density, identifying the reversible two-tissue compartment model and Logan graphical analysis as the optimal quantification methods [20]. A study of 54 healthy midlife women, using graphic Logan plots, revealed progressively higher distribution volume ratios (DVR) over the menopause transition in estrogen-regulated networks, with significant differences in pituitary, as well as posterior cingulate cortex and caudate [21]. Effects were independent of age, plasma E2 and sex hormone-binding globulin (SHBG) levels [21]. Additionally, case reports noted ^18^F-FES binding in pituitary, and non-specific binding in white matter of breast cancer patients and postmenopausal controls [22, 23].

To enhance the usability of ^18^F-FES PET for brain studies, simplified image analysis methods are desirable. The gold-standard approach for receptor quantification is the use of dynamic acquisition of PET data and analysis with methods such as kinetic modeling with an input function, or Logan plots to derive distribution volume ratios (DVRs) [24] or measures of non-displaceable binding potential (BP_ND_) [25]. For clinical applications, however, the SUVR method is favored due to its shorter scan duration, absence of need for arterial cannulation or dynamic imaging, and its computational simplicity [26].

Herein, we examined associations between DVR measures from dynamic ^18^F-FES PET scans and data collected in static modes for SUVR analyses in healthy midlife women, to determine the optimal SUVR time frame yielding comparable results to DVR measures. Our goal was to assess whether brain ER expression could be estimated from a single, late-scan static PET image showing high consistency with DVRs. The pituitary was our main target region of interest (ROI), given consistent evidence for specific binding in this region [15, 16]. For exploratory purposes, we also examined ER-rich hypothalamus, thalamus, hippocampus, amygdala, caudate, posterior cingulate cortex, middle and inferior frontal cortex [27–30].

## Methods

This is a natural history, non-interventional study of 55 clinically and cognitively normal midlife women at different endocrine stages, including approximately equal proportions of premenopausal (PRE, standardized to midcycle), perimenopausal (PERI), and postmenopausal (POST) participants. Participants were included in a previous publication evaluating brain ^18^F-FES DVRs [21], along with an additional postmenopausal participant whose laboratory data became available after the earlier analysis. This study extends analysis to SUVR data collected in static modes.

Participants were recruited at Weill Cornell Medicine (WCM) between 2021 and 2024 from multiple community sources, including individuals interested in research participation, family members and caregivers of impaired patients at our institution, and by word of mouth [31–34]. All gave written informed consent to participate in this ^18^F-FES PET study, which was approved by the WCM Institutional Review Board. Use of ^18^F-FES was carried out under WCM Radioactive Drug Research Committee and National Cancer Institute (NCI) Investigational New Drug (IND) #146,703 approval.

All participants underwent clinical examinations including medical history, neurological exams, neuropsychological testing, blood analysis including genetics and sex steroid hormones, multi-modal MRI and ^18^F-FES PET imaging. Participants were 40–65 year-old women with *≥* 12 years of education and a diagnosis of normal cognition per physician’s assessment, with Montreal Cognitive Assessment (MoCA) scores *≥* 26 and cognitive test performance within normative values for age and education [31–34]. Pre-established exclusion criteria included: (i) any significant neurological disease, such as dementia, normal pressure hydrocephalus, brain tumor, progressive supranuclear palsy, seizure disorder, subdural hematoma, multiple sclerosis, or history of significant head trauma followed by persistent neurologic deficits or known structural brain abnormalities; (ii) any significant psychiatric disease, such as major depression, bipolar disorder, schizophrenia, or psychotic features; (iii) T2 and/or FLAIR MRI brain scan evidence of infarction, lacunes or demyelination disease; (iii) systemic illnesses, unstable medical conditions or major medical complications such as treatment for neoplastic disease, unmanaged cardiovascular disease, diabetes, renal or liver disorder; (iv) history of drug or alcohol dependence; (v) current use of psychoactive medications (e.g. benzodiazepines, cholinesterase inhibitors, psychostimulants, etc.) or investigational agents; (vi) contraindications to MRI or PET imaging. Additional exclusion criteria included: (vii) history of oophorectomy or hysterectomy; (viii) use of hormonal therapy; (ix) active pregnancy.

Menopausal status was based on the Stages of Reproductive Aging Workshop (STRAW) criteria [35] with hormone laboratory assessments as supportive criteria (premenopause: regular cycle; perimenopause: no menses in the past 3–11 months; postmenopause: no menses for the past *≥* 12 months) [35]. Plasma E2, progesterone, follicle stimulating hormone, luteinizing hormone, SHBG, and testosterone levels were measured by a commercial laboratory (Boston Heart Diagnostics, Framingham, MA).

### Brain Imaging

#### Acquisition

All participants received MRI and PET scans following standardized protocols [31–34]. Scans were performed on consecutive days, except for 9 participants who completed FES an average of 0.8 *±* 1.9 months of MRI.

##### Volumetric MRI

3D volumetric T_1_-weighted MRI [BRAVO; 1 × 1 × 1 mm resolution, 8.2 ms repetition time (TR), 3.2 ms echo time (TE), 12° flip angle, 25.6 cm field of view (FOV), 256 × 256 matrix with ARC acceleration] were acquired on a 3.0 T MR750 Discovery scanner (General Electric, Waukesha, WI) using a 32-channel head coil in a single imaging session.

##### ^18^F-Fluoroestradiol PET imaging

16α-[^18^F]fluoro-17β-estradiol (^18^F-FES) was prepared by the WCM PET Radiochemistry Group using established methods for synthesis and quality assurance [36, 37]. ^18^F-FES scans were acquired using a Siemens BioGraph mCT 64-slice PET/CT scanner [70 cm transverse FOV, 16.2 cm axial FOV, voxel size 1.0 mm] operating in 3D mode. All scans were performed after a 4-hour fasting to decrease biliary uptake. One hour before PET imaging, an antecubital venous catheter was positioned for tracer injection. No arterial blood sampling was performed. Participants laid down on the scanner bed with eyes closed and ears unplugged, in the quiet and dimly lit scan room. Following a low-dose CT scan, a dose of approximately 6 mCi (222 MBq) of ^18^F-FES was infused intravenously in a volume of 20 mL isotonic phosphate buffered saline containing less than 15% of ethanol by volume over 2 min. Dynamic imaging was performed for 90 min, and consisting of 30 frames: 4 × 15, 4 × 30, 3 × 60, 2 × 120, 5 × 240, 12 × 300 s. All participants completed the full 90 min dynamic PET acquisition. All images were corrected for attenuation, scatter and radioactive decay.

#### Image analysis

Image processing was performed using a semi-automated pipeline [31–34]. The T_1_-weighted images were segmented using SPM Segment implemented in SPM12 [38] running on Matlab 2021 (MathWorks; Natick.MA), which included spatial normalization by high-dimensional warping (DARTEL) and the creation of warp-transform images [38]. ^18^F-FES dynamic images were motion-corrected by first creating a mean image of early frames from 1 to 8 min and then using that as an anchor for all frames within-modality using the surface-fitting Normalized Mutual Information (NMI) algorithm. ^18^F-FES image co-registration to the T_1_-weighted image was accomplished using the mean image of early frames from 1 to 8 min using NMI, with the co-registration parameters then applied to all frames of the motion-corrected dynamic image. The spatial transformation from the previous DARTEL operation was then applied to all motion-corrected co-registered ^18^F-FES frames.

##### Target regions

As ^18^F-FES selectively binds ERα [39], and tracer uptake in white matter is affected by non-specific binding [20, 21], we focused on predominantly gray matter regions with high ERα expression. The pituitary was our target ROI, given evidence for fully specific binding in this region [15, 16, 20]. ER-rich hypothalamus, thalamus, hippocampus, amygdala, caudate, posterior cingulate cortex, middle and inferior frontal cortex were also examined [27–30].

##### Reference region

Our approach for developing a suitable reference region ROI was previously published [21]. Briefly, we chose the cerebellum as the anatomical reference region based on evidence that it is generally free of, or minimally expresses ERα [28–30, 40–42]. This selection was further supported by kinetic studies in non-human primates, which validated the cerebellum as an appropriate reference region [19]. Given evidence that ERβ, the predominant ER subtype in the cerebellum, and GPER-1 are expressed in the innermost portion of cerebellar white matter and adjacent gray matter (corresponding to human middle cerebellar peduncle, culmen, arbor vitae, dentate nucleus, and medullary cortex) [28–30, 40, 42], we developed a probabilistic cluster-based cerebellar ROI restricted to the outermost portion of cerebellar crus II gray matter. We then used voxel-based machine learning with intensive iterative data resampling implemented in NPAIRS (nonparametric prediction, activation, influence, and reproducibility resampling) [43] to further restrict the ROI to the inferior portion of cerebellar crus II, which showed invariant tracer uptake across menopause classes, which is a prerequisite for normalization [44].

#### Kinetic modeling and simplified analysis

Parametric binding potential (BPnd) images were generated using the pixel-wise modeling tool (PXMOD) implemented in PMOD v4.1 (PMOD Technologies). Graphic Logan plots [24] were used to derive binding potentials (BPnd) using the time-activity curve (TAC) of a custom-made cerebellar gray matter reference region, as previously described [21]. Only voxels with BPnd > 0 were retained. Static summed ^18^F-FES PET images were generated for five 20-min increments (30–50, 40–60, 50–70, 60–80, and 70–90 min post-injection) using custom-made scripts.

ROI placement and sampling were conducted using select ROIs from the anatomical labeling atlas (AAL3) [45] implemented in WFU PickAtlas, applied to each participant’s PET. Selected ROIs were gray matter-based with placement confirmed by visual inspection. The pituitary ROI was generated as a probabilistic volume of interest (VOI) of approximately 2 mm radius, and fitting was confirmed on the coregistered anatomical MRI by two expert raters (MN, VB). ROIs were applied to the BPnd images to calculate distribution volume ratios (DVR), where DVR = 1 + BPnd [24, 25]. SUVRs in each target ROI were calculated for each 20-min time frame by normalizing uptake to the reference cerebellar ROI.

### Statistical analysis

Analyses were performed in SPSS v.28, R v.4.2 and SPM12. Clinical measures were examined with general linear models or chi-squared tests as appropriate. Results are considered significant at *P* < 0.05 throughout.

#### Correlation and reliability analysis

Regional DVR and SUVR values over different time frames were examined using Pearson’s *r* correlation coefficients and Intra-class Correlation Coefficients (ICC) with Cronbach’s Alpha as reporting criterion. Correlation heatmaps and scatterplots were employed to visualize relationships between DVR and SUVR on a regional basis.

#### Data-driven variable selection procedures

Stepwise linear regression and Least Absolute Shrinkage and Selection Operator (LASSO) modeling were used to identify the optimal SUVR intervals for each brain region. For variable selection procedures retaining multiple time frames as independently meaningful, we constructed univariate models for each SUVR time frame and selected the measure with the smallest Akaike Information Criterion (AIC) as the optimal time interval for that given ROI.

### Sensitivity analysis

#### Analysis of menopause status effects

- To test whether associations between DVR and SUVR measures were modified by menopause status, linear regression modeling was used to quantify the effects of menopause status, SUVR time frame, and their interaction when predicting the DVR measure for each ROI. A separate model was created for each pairwise combination of time intervals and brain regions.
- For explanatory purposes and to assist in the interpretation of findings, we examined differences across menopause statuses within the sets of SUVR images demonstrating the highest consistency with DVR measures. As we previously reported progressively higher DVR measures from the premenopausal to the postmenopausal group [21], in this analysis, we capitalize on these two distinct stages to examine the magnitude of signal differences reflected by SUVR metrics. Using an ROI approach, standardized marginal mean differences between groups were calculated for each interval-specific SUVR measures and Cohen’s d effect sizes were used to identify the time frames that maximized detection of group differences, adjusting for age and plasma SHBG.

#### Associations of ER density and cognitive performance

Based on our previous findings that higher DVRs in amygdala, hippocampus, frontal and posterior cingulate cortex were associated with lower scores on immediate and delayed recall in the Logical Memory test [21], we examined whether SUVRs in the same regions showed comparable associations. We also tested associations with global cognition (MoCA scores). Linear regression models were developed with standardized cognitive test scores as the outcomes and ER density as the primary exposure, adjusting for age and education, at *P* < 0.05.

## Results

Participant characteristics are shown in Table 1. Fifty-five participants were examined, including 18 PRE, 18 PERI, and 19 POST women. The study cohort was in good general health, with only a small percentage of individuals with clinically managed hypertension, hypercholesterolemia (cholesterol > 240 mg/dL), and/or type 2 diabetes (*≤* 1%) (Table 1).

**Table 1:** Participant characteristics

*N*	55
Age, years	50(6)
Education, years	17(2)
Race, % white	82
MoCA score, unitless	28(2)
Hypertension, % positive	1
Hypercholesterolemia, % positive	0.07
Diabetes, % positive	0.04
Smoking, % never smoker	76
Menopause status, n (pre-, peri-, post-menopause)	18, 18, 19

Values are means (standard deviation) or percentages (%) as indicated. Abbreviations: MoCA, Montreal Cognitive Assessment

### Correlation analysis

**Fig. 1:**
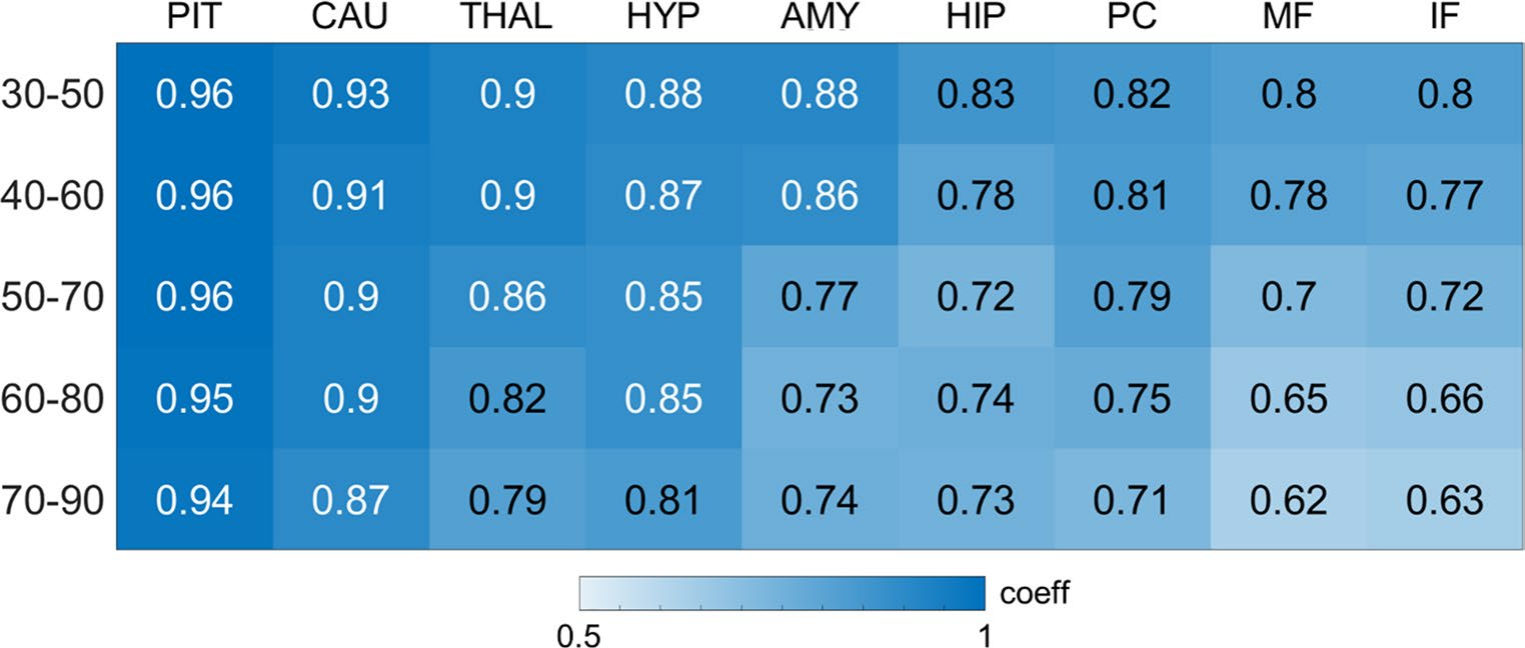
Heatmap of regional correlations between distribution volume ratios (DVR) and standardized uptake value ratios (SUVR) at different time frames. Heat maps showing associations between ^18^F-FES DVR and SUVR across different time frames (30–50, 40–60, 50–70, 60–80 and 70–90 min post-injection) for each brain region. Pearson’s r correlation coefficients are significant at *P* < 0.001 and displayed on a color-coded scale, with darker blue indicating stronger correlations and lighter blue indicating weaker correlations. Our main target was the pituitary. Additional ER-rich regions are provided for exploratory purposes. Abbreviations: AMY, amygdala; CAU, caudate; HIP, hippocampus; HYP, hypothalamus; IF, inferior frontal; MF, middle frontal; PC, posterior cingulate; PIT, pituitary; THAL, thalamus

**Fig. 2:**
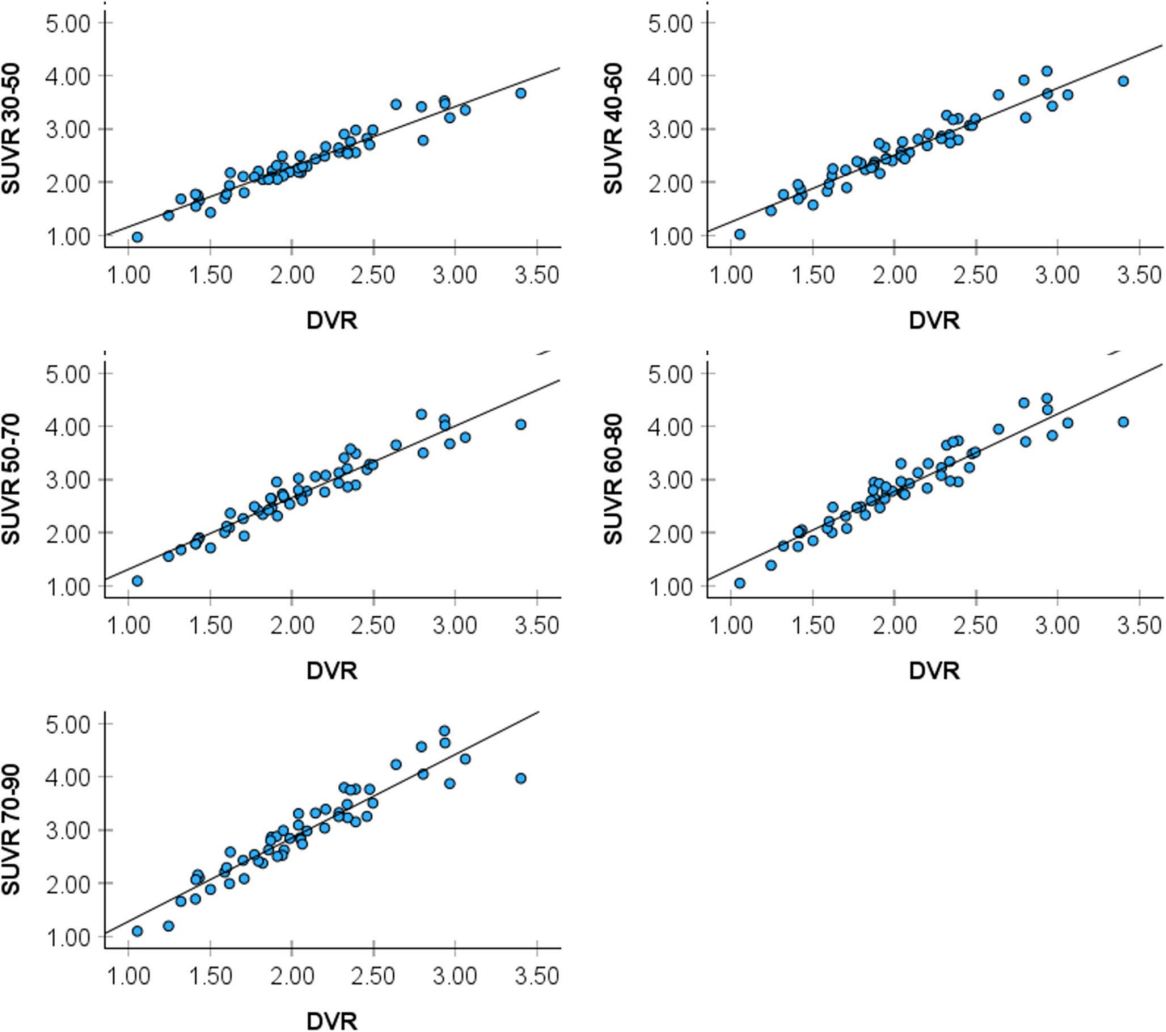
Distribution volume ratios (DVR) vs. standardized uptake value ratios (SUVR) at different time frames in the pituitary region. Scatter plots showing associations between DVR and SUVR at five 20-minute intervals in the pituitary ROI

Pearson’s r correlation coefficients across different brain regions are shown in Fig. 1. For the pituitary, all coefficients were statistically significant at all time points (*P* < 0.001) yet descriptively higher in the earlier (30–50 and 40–60 min) vs. later time frames. This effect is exemplified in Fig. 2. For exploratory ROIs, there was a progressive decrease in correlation values from the 30–50 min to the 70–90 min time frames (Fig. 1). Generally, the 30–50 and 40–60 min frames showed the strongest correlations with DVR measures.

### Reliability analysis

Results from reliability analysis are reported in Table 2. ICC analysis indicated a generally high agreement between pituitary DVR and SUVR measures across all time frames (*P* < 0.001). The 30–50 min frame displayed the highest ICCs, followed by the 40–60 min interval. As with the correlation analysis, agreement tended to diminish from earlier to later time frames, with the 70–90 min frame exhibiting the lowest ICCs. A similar pattern was observed for exploratory ROIs (Table 2).

**Table 2:** Reliability analysis: intraclass correlation coefficients

	30–50		40–60		50–70		60–80		70–90	
	ICC	*p*	ICC	*p*	ICC	*p*	ICC	*p*	ICC	*p*
Pituitary	0.973	< 0.001	0.960	< 0.001	0.948	< 0.001	0.927	< 0.001	0.906	< 0.001
Amygdala	0.872	< 0.001	0.866	< 0.001	0.831	< 0.001	0.813	< 0.001	0.804	< 0.001
Caudate	0.646	< 0.001	0.624	< 0.001	0.619	< 0.001	0.614	< 0.001	0.610	< 0.001
Hippocampus	0.827	< 0.001	0.793	< 0.001	0.763	< 0.001	0.762	< 0.001	0.756	< 0.001
Hypothalamus	0.767	< 0.001	0.782	< 0.001	0.809	< 0.001	0.772	< 0.001	0.741	< 0.001
Inferior frontal	0.718	< 0.001	0.695	< 0.001	0.660	< 0.001	0.611	< 0.001	0.588	< 0.001
Middle frontal	0.672	< 0.001	0.645	< 0.001	0.591	< 0.001	0.543	< 0.001	0.510	< 0.001
Posterior cingulate	0.803	< 0.001	0.788	< 0.001	0.779	< 0.001	0.766	< 0.001	0.733	< 0.001
Thalamus	0.905	< 0.001	0.900	< 0.001	0.879	< 0.001	0.860	< 0.001	0.836	< 0.001

Intraclass correlation coefficients (ICC) for distribution volume ratio (DVR) versus standardized uptake value ratio (SUVR) in target regions are displayed by SUVR time frame, with corresponding P values. Our main target was the pituitary. Additional ER-rich regions are provided for exploratory purposes

### Automated selection procedures

Both stepwise linear regression and LASSO analyses identified the 30–50 min time frame as demonstrating the highest association with pituitary DVR measures (Tables 3 and 4). For exploratory ROIs, on stepwise linear regressions, the 30–50 min time frame demonstrated the highest associations with DVR measures in all regions except thalamus and middle frontal ROIs, where the 40–60 min time frame provided the best fit (Table 3). The combination of LASSO analysis with AIC criteria showed similar results, with the 30–50 min SUVR time frame being selected as providing the best fit relative to DVR measures across all regions except for the thalamus, where the 40–60 min interval proved most suitable (Table 4).

**Table 3:** Stepwise linear regression analysis

	Selected SUVR time frame	Estimate	*p*-value
Pituitary	30–50	0.83 (0.34, 1.32)	< 0.001
Amygdala	30–50	0.53 (0.45, 0.6)	< 0.001
Caudate	30–50	0.26 (0.23, 0.29)	< 0.001
Hippocampus	30–50	0.56 (0.35, 0.77)	< 0.001
Hypothalamus	30–50	0.23 (0.16, 0.31)	< 0.001
Inferior frontal	30–50	0.46 (0.31, 0.61)	< 0.001
Middle frontal	40–60	0.91 (0.6, 1.22)	< 0.001
Posterior cingulate	30–50	0.42 (0.34, 0.51)	< 0.001
Thalamus	40–60	0.32 (0, 0.64)	0.053

The best standardized uptake value ratio (SUVR) time frame selected by stepwise regression analysis is reported for each region, with corresponding estimates (95% confidence interval) and p-values. Our main target was the pituitary. Additional ER-rich regions are provided for exploratory purposes

**Table 4:** LASSO analysis

	Selected SUVR time frame	AIC
Pituitary	30–50	**−55.30153**
	50–70	−50.51647
	70–90	−33.54014
Amygdala	30–50	**−250.8217**
	40–60	−243.0585
Caudate	30–50	**−289.5557**
Hippocampus	30–50	**−224.7702**
	40–60	−212.5407
	50–70	−202.8510
	60–80	−205.0241
	70–90	−204.0196
Hypothalamus	30–50	**−253.5364**
	40–60	−250.2595
	60–80	−240.6651
	70–90	−230.9286
Inferior frontal	30–50	**−271.4635**
	40–60	−265.4747
	60–80	−245.5704
Middle frontal	30–50	**−280.2428**
	40–60	−274.6222
	50–70	−261.4468
	70–90	−249.7366
Posterior cingulate	30–50	**−189.2783**
	50–70	−181.3789
	70–90	−166.1763
Thalamus	30–50	−229.3227
	40–60	**−230.4417**

Standardized uptake value ratio (SUVR) time frames retained by Least Absolute Shrinkage and Selection Operator (LASSO) analysis are reported for each region, with corresponding Akaike Information Criterion (AIC). The lowest AIC value for each region, indicating the best time frame, is reported in bold. Our main target was the pituitary. Additional ER-rich regions are provided for exploratory purposes

### Sensitivity analysis

#### Effects of menopause status

**Fig. 3:**
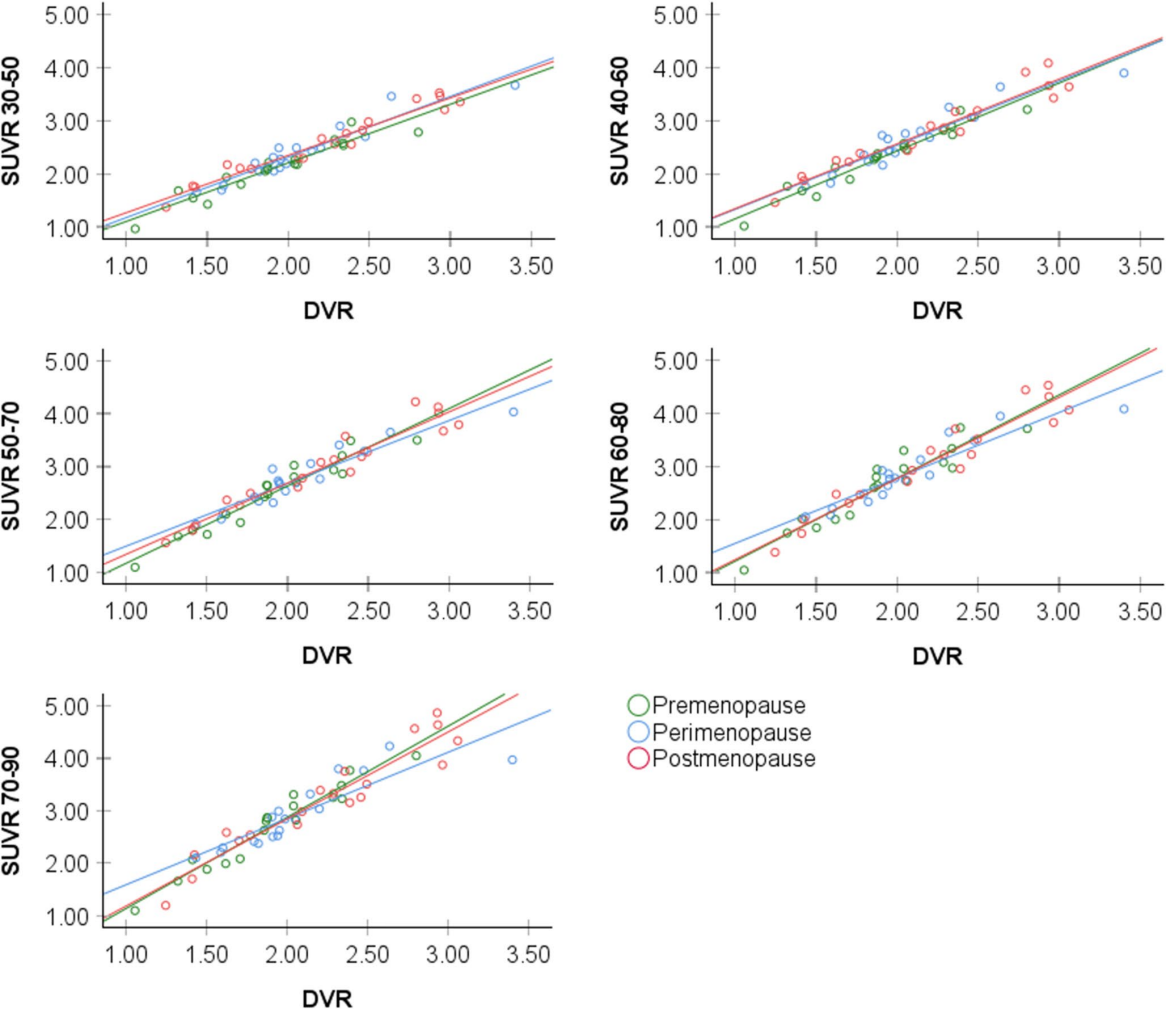
Scatter plots of distribution volume ratios (DVR) vs. standardized uptake value ratios (SUVR) in the pituitary region by menopause status. Scatter plots showing associations between pituitary DVR and SUVR over five 20-minute intervals by menopause status

There were no significant interaction effects between SUVR measures at different time frames and menopause status when predicting pituitary DVR values (Fig. 3) or for any given exploratory ROI. This suggests that the selected SUVR time intervals are applicable across menopausal groups.

**Fig. 4:**
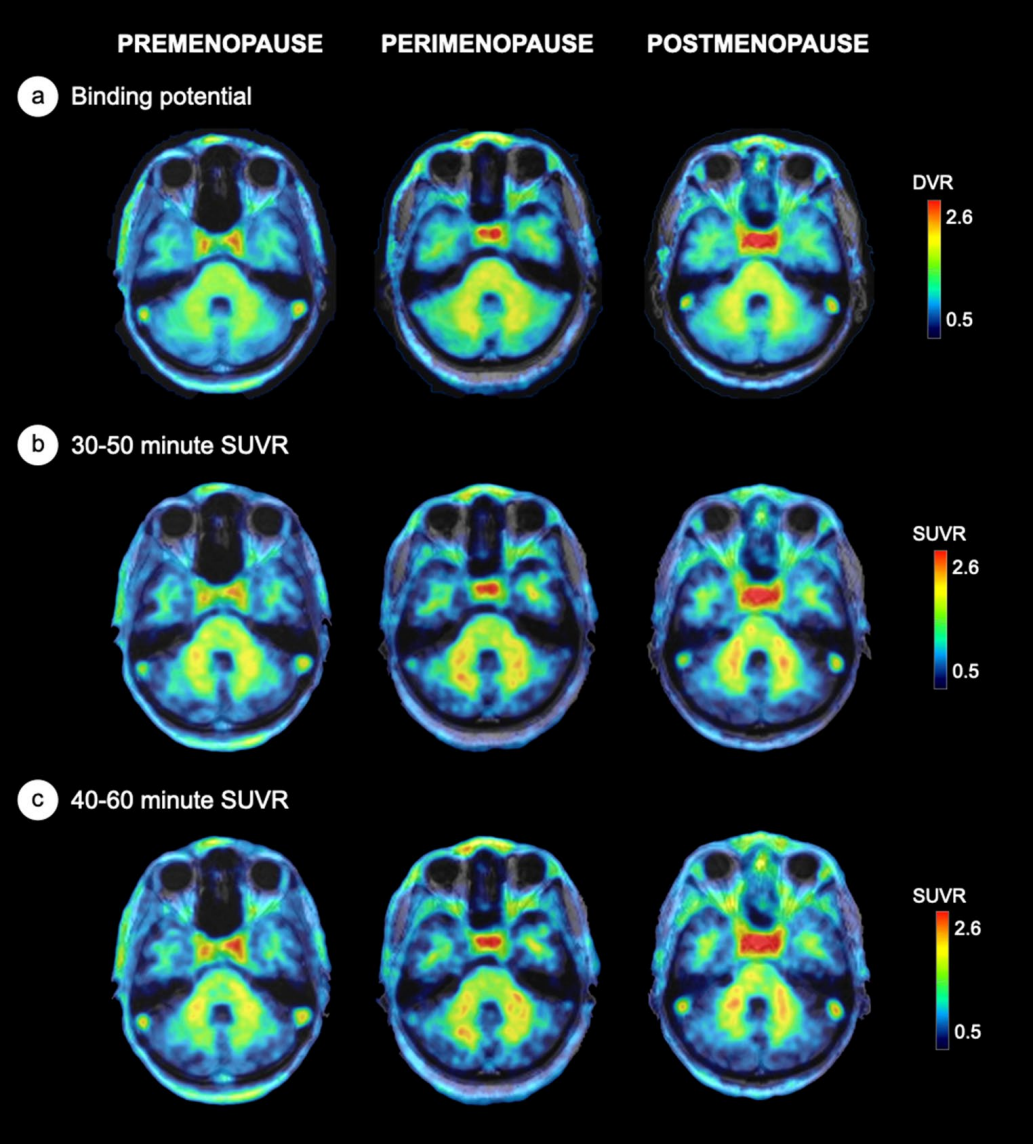
Menopausal stage differences in [¹⁸F]Fluoroestradiol (FES) PET uptake in the pituitary region. Representative parametric [^18^F]Fluoroestradiol (FES) PET images from three participants—premenopausal, perimenopausal, and postmenopausal (left to right)—are shown. ^18^F-FES data are overlaid on coregistered structural MRI: (**a**) Binding potential (BPnd), used to derive distribution volume ratios (DVR); (**b**) standardized uptake value ratio (SUVR) from 30 to 50 min post-injection; (**c**) SUVR from 40 to 60 min post-injection. All methods show a consistent pattern of increased ^18^F-FES signal in the pituitary with advancing menopausal stage.

As various analytical approaches identified the 30–50 min SUVR time frame as optimal for the pituitary and most exploratory regions, followed closely by the 40–60 min interval, these two intervals were carried into examination of menopausal group effects. We previously reported progressively higher DVR from the premenopausal to the postmenopausal stage in this cohort [19]. In the present analysis, both 30–50 and 40–60 min time frames demonstrated higher pituitary SUVR in the postmenopausal compared to the premenopausal group, consistent with DVR results, yielding a medium to large effect size of d = 0.927 for the 30–50 min interval and d = 0.911 for the 40–60 min interval (Table 5). Exploratory ROIs also yielded medium to large effect sizes when differentiating between postmenopausal and premenopausal groups (Table 5). This effect is exemplified in Fig. 4.

**Table 5:** Effect size of SUVR measures in differentiating menopausal groups

	30–50 min	*p*-value	40–60 min	*p*-value
Pituitary	0.927 (1.622, 0.232)	0.021	0.911 (1.606, 0.217)	0.023
Amygdala	0.852 (1.543, 0.161)	0.036	1.063 (1.766, 0.361)	0.007
Caudate	0.370 (1.044, −0.304)	0.514	0.373 (1.047, −0.301)	0.508
Hippocampus	0.868 (1.561, 0.176)	0.032	0.828 (1.518, 0.138)	0.043
Hypothalamus	0.521 (1.199, −0.157)	0.272	0.558 (1.237, −0.121)	0.226
Inferior Frontal	1.031 (1.732, 0.331)	0.009	0.978 (1.676, 0.281)	0.014
Middle Frontal	0.976 (1.673, 0.278)	0.014	0.95 (1.646, 0.254)	0.017
Posterior Cingulate	1.202 (1.913, 0.49)	0.002	1.193 (1.904, 0.482)	0.002
Thalamus	0.638 (1.32, −0.045)	0.146	0.753 (1.44, 0.066)	0.072

Cohen’s d effect size for postmenopausal vs. premenopausal groups at 30–50 and 40–60 min post-injection, where d = 0.2 reflects a small effect size, d = 0.5 a medium effect size, and d ≥ 0.8 a large effect size. Our main target was the pituitary. Additional ER-rich regions are provided for exploratory purposes

#### Associations of ER density with cognition

Results are reported in Table 6. Negative associations were observed between 30 and 50 min SUVRs in hippocampus, amygdala, and frontal cortex and delayed recall scores (multivariable-adjusted *P* ≤ 0.047). In addition, hippocampal SUVR was negatively associated with immediate recall (multivariable-adjusted *P* = 0.042) and global cognition scores (multivariable-adjusted *P* = 0.044). A negative association was observed between 40 and 60 min hippocampal SUVR and delayed recall scores (multivariable-adjusted *P* = 0.015).

**Table 6:** Associations of regional SUVR measures with cognitive performance

	Logical memory, immediate recall	*p*	Logical memory, delayed recall	*p*	Global cognition	*p*
***30–50 min***						
Amygdala	−0.212	0.136	−0.334	**0.017**	−0.187	0.189
Hippocampus	−0.286	**0.042**	−0.396	**0.004**	−0.284	**0.044**
Inferior Frontal	−0.189	0.184	−0.253	***0.073***	−0.122	0.392
Middle Frontal	−0.255	0.070	−0.279	**0.047**	−0.113	0.428
Posterior Cingulate	−0.046	0.750	−0.216	0.128	−0.140	0.326
***40–60 min***						
Amygdala	−0.157	0.270	−0.266	***0.060***	−0.226	0.110
Hippocampus	−0.233	***0.099***	−0.338	**0.015**	−0.256	***0.070***
Inferior Frontal	−0.128	0.371	−0.190	0.181	−0.097	0.496
Middle Frontal	−0.194	0.172	−0.215	0.130	−0.073	0.609
Posterior Cingulate	0.002	0.988	−0.163	0.254	−0.077	0.592

Partial correlation coefficients and associated P values from linear regression models. Significant P values are in bold, trends are in italics

## Discussion

This study sought to examine the suitability of simplified image analysis for brain ^18^F-FES PET imaging by comparing regional DVR and SUVR across five 20-minute increments beginning 30 min post-injection. We focused on the pituitary gland as our target, given that it is the most consistently reported site of ^18^F-FES-specific binding across species [15–17, 20, 21, 46]. Additional ER-rich regions were examined for exploratory purposes. Automated variable selection procedures identified the 30–60 min window as yielding the SUVR measures most closely associated with DVR, thus balancing study feasibility and physiological validity. In sensitivity analyses, this time window was also suitable for detection of menopause status effects and associations with cognitive performance.

While human brain ^18^F-FES PET studies remain scarce, animal research has provided valuable insights into the ligand’s performance in the brain, consistently highlighting the pituitary as the primary uptake site [15–17]. Kinetic analyses in rats, including Logan plot modeling, showed high pituitary binding, surpassing levels observed in cortical and limbic areas [15, 16]. In inhibition studies, co-injection of 17β-estradiol almost abrogated pituitary uptake, indicating specific binding [15, 16]. While the posterior pituitary is partially protected by the blood-brain barrier (BBB) due to its mixed vascular features, the anterior pituitary lies outside the BBB, allowing direct access to circulating tracers, which may account for its high uptake. However, preclinical data indicate that ^18^F-FES uptake is not restricted by the BBB. Influx constant values are comparable to those of other PET ligands [16], and specific uptake has been observed in the hypothalamus—a region generally protected by the BBB [15, 16, 18]. Lower, yet measurable ^18^F-FES uptake was also reported in the striatum, cortex, hippocampus, bed nucleus of the stria terminalis (BNST), and amygdala [15, 16]. Whether uptake in low-uptake regions is specific remains to be determined, as only two studies—both ex vivo and with small sample sizes ≤ 4 rats per group- have addressed this question [15, 16]. Results were mixed, with some regions showing no significant blocking with 17β-estradiol co-injection [15, 16] while others showed significant differences in tracer uptake [16]. Additionally, the translatability of rodent findings to humans is limited. Unlike rats, humans produce SHBG, which protects ^18^F-FES from rapid clearance. Faster metabolism in rats may limit tracer availability in regions with low ER density, whereas in humans, ^18^F-FES PET may remain effective [16]. This suggests that tracers with higher ER affinity may be needed in rodent studies, though not necessarily for human imaging.

Complementary evidence comes from non-human primates. In rhesus macaque brains, full kinetic modeling with a metabolite-corrected arterial input function and pharmacological blockade confirmed specific ^18^F-FES binding in the pituitary [19]. The cerebellum was identified as a suitable reference region, showing no reversible binding after estradiol pre-treatment [19]. No other brain regions exhibited specific ^18^F-FES binding [19]. However, the sample size was limited to two monkeys, one male and one female, and for safety reasons, the estradiol blocking dose used was potentially subtherapeutical [19], about one-third of the dose employed in rodent studies [16].

To date, only one study has specifically investigated ^18^F-FES kinetics in the human brain [20]. In a small sample of seven healthy postmenopausal women, kinetic modeling with metabolite corrected arterial input function identified the two-tissue compartment model (with a fixed K₁/k₂ ratio) and the Logan graphical analysis as the preferred methods [20]. Consistent with preclinical studies, the highest uptake was observed in the pituitary [20]. Importantly, the influx constant was comparable across brain regions, supporting animal data suggesting that high pituitary binding is not solely attributable to its partial location outside the BBB [20]. The study also reported significant correlations between regional kinetic measures and SUV values measured at 80–90 min post-injection [20]. No alternative time frames were evaluated, and SUVRs were not calculated. Given our findings showing poorer reliability of later time frames such as 70–90 min, it would be interesting to explore whether earlier time frames might have yielded stronger associations with kinetic measures.

In a subsample of four participants, administration of an experimental ER degrader significantly reduced pituitary ER availability, while no clear changes were observed in other brain regions [20]. As the authors noted, the very small sample size limited statistical power, potentially obscuring additional binding sites. Moreover, the study used an ER degrader instead of “cold” estradiol typically used in ^18^F-FES inhibition studies, which may have further reduced blocking efficacy [20]. Larger, rigorously controlled kinetic studies are warranted to confirm regional binding specificity besides the pituitary.

Notably, oophorectomized rats exhibit increased pituitary and hypothalamic ^18^F-FES BPnd and distribution volume (Vt) [16], along with significantly increased pituitary SUV compared to controls [16, 17], highlighting the ligand’s sensitivity to hormonal status in those regions. Oophorectomized rats also exhibited higher ^18^F-FES uptake in pituitary and hypothalamus (~ 70%), BNST (87%), amygdala (47%), and midbrain (27%) compared to proestrous rats [16]. These results align with mechanistic studies showing increased ER mRNA levels [47] and immunoreactivity [48] in multiple brain regions post-oophorectomy—including hypothalamic subnuclei, medial preoptic area, bed nucleus of the stria terminalis, arcuate nucleus, and ventral premammillary nuclei [47–49] —and findings of ER rebound in aging female rats at the early menopausal stage [49]. Collectively, these findings support the feasibility of ^18^F-FES PET for brain imaging.

Increased brain ^18^F-FES ER expression post-menopause was also observed in human studies. A study of *de novo* breast cancer patients assessed pituitary uptake at 60 min post-injection, showing marginally higher SUVR in the postmenopausal compared to the premenopausal patient groups [17]. However, the study had an uneven sample size, with 9 premenopausal and 22 postmenopausal women, and PET imaging in the premenopausal group was not standardized according to the menstrual cycle [17]. Technical limitations included the reliance on tracer uptake for pituitary delineation instead of MRI-guided tracing, and the normalization of the pituitary signal to that in the frontal cortex [17] –a known ER site [27–30]– which may have hindered detection of menopausal status effects. This study did not examine other brain regions. Our recent brain ^18^F-FES PET study, involving a prospective cohort of 54 healthy midlife women segmented into three size-matched groups by menopausal status, addressed some of these limitations [21]. Using MRI-guided ROIs along with graphic Logan plots using the cerebellum as the reference region, the postmenopausal group exhibited significantly higher pituitary DVR compared to the premenopausal group, with intermediate values in the perimenopausal group [21], adjusting by age, plasma E2 and SHBG. A similar pattern was also observed in posterior cingulate, caudate and frontal regions [21].

In the present analysis, we leveraged the same study cohort [21] to conduct a validation study of SUVR measurements in the pituitary. Various analytical techniques showed that SUVRs were generally consistent with DVRs, with a progressive decrease in agreement from earlier to later time frames. Such time-dependent variability is a known feature of SUVR methods [50], underscoring the importance of multi-time frame examinations as performed in this study. Data-driven selection procedures indicated that the 30–50 min window was optimal for the pituitary, closely followed by the 40–60 min interval. Both the 30–50 and 40–60 min timeframes were effective in detecting increased pituitary SUVRs from premenopausal to postmenopausal stages. Similar results were obtained for the additional ER-rich regions examined for exploratory purposes.

Overall, the combined 30–60 min range appears suitable for static brain ^18^F-FES acquisition. This is consistent with observations that ^18^F-FES rapidly accumulates in ER-expressing tissue within 20–30 min and returns to sub-physiologic levels within approximately 1 h post-injection [17, 51]. This time window could facilitate clinical application by accommodating inter-center technological variability, and simplifying protocols and routines, akin to the 30–60 min acquisition time used for brain ^18^F-FDG PET.

A possible drawback of the SUVR approach is its tendency to overestimate receptor binding relative to DVR, in a manner that increases over time from injection [26]. However, overestimation is more pronounced when computing the tissue to plasma concentration ratio than following normalization of regional radioactivity to that of a reference with low or no binding [26], as in this study. Quantitatively, in rodents, ovariectomy was associated with a 78% increase in pituitary ^18^F-FES uptake measured by Vt [16], and a 64–70% increase using SUV and SUVR measures [16, 17]. In our previous DVR study, the postmenopausal group exhibited 36% higher age-adjusted pituitary DVR compared to the premenopausal group [21], which is plausible for women undergoing spontaneous menopause. In the current study, the postmenopausal group exhibited 36% higher pituitary SUVR in the 30–50 min time frame and 37% higher SUVR in the 40–60 min time frame compared to the premenopausal group, adjusting for age. These data suggest that SUVR differences within these time frames are unlikely to be affected by overestimation bias.

The choice of a suitable reference region is crucial for DVR as well as SUVR measures. As described in [21], our reference ROI was restricted to the cerebellar crus II gray matter, based on preclinical and postmortem evidence that this part of the cerebellum does not express, or minimally expresses ERα [27–30, 42], consistent with the known selectivity of ^18^F-FES for ERα. This choice is further supported by kinetic brain ^18^F-FES PET studies in rhesus macaques, which identify the cerebellum as a suitable reference region [19]. Although estrogen plays an important role in cerebellar functions—such as balance, motor coordination, and cerebellar circuitry—these effects are primarily mediated by ERβ, not ERα [42]. Because ^18^F-FES binds more selectively to ERα, which is sparsely expressed in the cerebellum, this region is unsuitable for assessing ER density effects per se but is appropriate for use as a reference region. The development of ERβ-specific tracers will be essential for studying cerebellar estrogen function, particularly in the context of menopause-related changes.

Our cerebellar ROI was further optimized by means of supervised clustering algorithms (SCA) to ensure that tracer uptake was both low and invariant by exposure (e.g. menopause status) [21], which is a key prerequisite for tracer normalization [44]. SCA methods are commonly used to extract pseudo-reference regions for non-invasive quantification of PET tracers with low intracerebral uptake, such as activated microglia TSPO (translocator protein) ligands [52]. Moreover, SUVR usage is widely accepted for various brain radiotracers, such as amyloid tracers, both fluorinated and Pittsburgh Compound B (PiB) [53], or radiolabeled amino acids (e.g. ^18^F-fluorodopa and ^18^F-fluoroethyltyrosine). Another advantage of SUVR measures is that sources of variability such as injected dose and body mass cancel out. Nonetheless, it is possible that we may have underestimated ER density in the cerebellar ROI. This would however conservatively reduce power in detecting ER density effects, especially across menopause groups.

In sensitivity analyses, the 30–50 min window showed stronger associations with cognitive performance than the 40–60 min window in limbic and frontal regions. These results are consistent with previous DVR findings [21], showing associations between hippocampal, amygdala and frontal DVRs and memory scores. While DVR measures in the posterior cingulate were also associated with delayed recall scores, SUVRs at either time window were not (e.g., *P* = 0.128 for the 30–50 min window). This may reflect lower signal-to-noise ratios for SUVR in the posterior cingulate cortex or region-specific differences in SUVR reliability, warranting further investigation. A new finding in this study is the association between 30 and 50 min hippocampal SUVRs and MoCA scores.

Strengths of this study include the cohort of carefully screened clinically healthy midlife women at different menopausal stages, ages 40–65 years, with no comorbidities or incidental findings, with complete clinical exams, laboratory tests, menopause assessments, and brain imaging. Our exclusion criteria ensured absence of confounding factors such as cancer, oophorectomy/hysterectomy, and hormone therapy use, paired with correction for age and SHBG levels. From a methodological perspective, we used state-of-the-art ROIs to analyze both DVR and SUVR, alongside data-driven variable selection procedures and reliability analysis.

A main limitation of the ^18^F-FES ligand, partly due to its high lipophilicity, is its non-specific binding. Specifically, previous research indicated that tracer uptake in white matter could not fully blocked by the administration of cold tracer [22, 23], thus reflecting non-specific uptake. White matter regions were excluded from the current analysis, and our primary target was the pituitary gland, which has been shown to exhibit specific tracer uptake based on both preclinical and clinical studies [15–17, 20, 21].

Moreover, while further research is needed, our findings show ER density increases with menopausal status in pituitary as well as other regions. These results cannot be explained solely by nonspecific binding, which is by definition independent of menopause status or ER expression. If nonspecific binding were the primary factor, the sampled signal would be uniform across groups, confounding detection of group differences. As mentioned above, these results are also consistent with mechanistic studies indicating an ER rebound after the early phase of menopause transition [49] and in vivo evidence of regionally elevated ^18^F-FES PET uptake in oophorectomized rats [16, 17]. Nevertheless, given these limitations, we consider our findings in brain regions other than the pituitary to be preliminary and hypothesis-generating.

## Conclusion

Examination of quantitative and simplified methods for analysis of brain 18F-FES PET uptake identified the 30–60 min SUVR time frames as performing optimally relative to DVR measures, providing a practical method for quantifying relative pituitary tracer retention in clinical populations.

## Data Availability

The data generated and analyzed during the current study are available from the corresponding author to qualified investigators, upon reasonable request.
